# Gait characteristics of subjects with chronic fatigue syndrome and controls at self-selected and matched velocities

**DOI:** 10.1186/1743-0003-5-16

**Published:** 2008-05-27

**Authors:** Lorna Paul, Danny Rafferty, Leslie Wood, William Maclaren

**Affiliations:** 1 Nursing and Health Care – Faculty of Medicine, University of Glasgow, Glasgow, UK; 2 School of Health & Social Care, Glasgow Caledonian University, Glasgow, UK; 3 School of Life Sciences, Glasgow Caledonian University, Glasgow, UK; 4 School of Engineering and Computing, Glasgow Caledonian University, Glasgow, UK

## Abstract

**Background:**

Gait abnormalities have been reported in individuals with Chronic Fatigue Syndrome (CFS) however no studies exist to date investigating the kinematics of individuals with CFS in over-ground gait. The aim of this study was to compare the over-ground gait pattern (sagittal kinematics and temporal and spatial) of individuals with CFS and control subjects at their self-selected and at matched velocities.

**Methods:**

Twelve individuals with CFS and 12 matched controls participated in the study. Each subject walked along a 7.2 m walkway three times at each of three velocities: self-selected, relatively slow (0.45 ms^-1^) and a relatively fast (1.34 ms^-1^). A motion analysis system was used to investigate the sagittal plane joint kinematics and temporal spatial parameters of gait.

**Results:**

At self-selected velocity there were significant differences between the two groups for all the temporal and spatial parameters measured, including gait velocity (P = 0.002). For the kinematic variables the significant differences were related to both ankles during swing and the right ankle during stance. At the relatively slower velocity the kinematic differences were replicated. However, the step distances decreased in the CFS population for the temporal and spatial parameters. When the gait pattern of the individuals with CFS at the relatively fast walking velocity (1.30 ± 0.24 ms^-1^) was compared to the control subjects at their self-selected velocity (1.32 ± 0.15 ms^-1^) the gait pattern of the two groups was very similar, with the exception of both ankles during swing.

**Conclusion:**

The self-selected gait velocity and/or pattern of individuals with CFS may be used to monitor the disease process or evaluate therapeutic intervention. These differences may be a reflection of the relatively low self-selected gait velocity of individuals with CFS rather than a manifestation of the condition itself.

## Background

Chronic Fatigue Syndrome (CFS) is thought to have a population prevalence of around 0.5% [1]. Although CFS is a recognised clinical condition the aetiology and pathology remain uncertain and consequently there is no specific diagnostic test for CFS. Recent research, however, has reported alterations in the expression of 16 specific genes in those with CFS, suggesting a pathology involving T cell activation and irregularities in neuronal and mitochondrial function [2].

Although there is no mortality associated with the condition, a recent systematic review suggested that only around seven percent of sufferers experience a full recovery whilst just under 40% report some improvement over time [3]. Thus the effects of CFS can lead to significant and prolonged functional disability.

Whilst there is a clinical impression that those with CFS display a different gait pattern compared to their healthy peers there is a paucity of studies investigating the effect of CFS on gait. Boda et al [4] examined the gait pattern of 11 individuals with CFS as they walked on a treadmill at three different velocities 0.45 ms^-1^, 0.89 ms^-1 ^and 1.34 ms^-1^. The researchers identified that those with CFS displayed significant differences in a number of the kinematic variables compared to the healthy control group. Specifically they reported reduced knee flexion during stance and swing at the slower velocity (0.45 ms^-1^) and increased hip flexion during stance and swing phases at the faster velocity (1.34 ms^-1^). Whilst it is interesting to compare the kinematics of gait at a number of different velocities the study utilised a treadmill for walking and the debate continues as to whether the gait pattern during treadmill walking is indeed comparable to over-ground walking [5-7].

Paul et al. [8] used a pressure sensitive, instrumented walkway to examine the temporal and spatial gait parameters of individuals with and without CFS before, and at intervals up to 24 hours after a 15 minute period of exercise. The results of the study suggested that, overall, there was a significant difference between the two groups with respect to step distance and step time on both right and left sides, single support time on the right, velocity and cadence. Although the data suggested changes in the temporal and spatial parameters at preferred walking pace these could have been influenced by the reduced over-ground walking velocity in individuals with CFS rather than changes due to the condition.

The aim of this study was to compare the sagittal joint kinematics and the temporal and spatial parameters of gait during over-ground walking at three velocities: self-selected, 0.45 ms^-1 ^and 1.34 ms^-1 ^between individuals with CFS and a control group. The two latter velocities correspond to the velocities previously examined [4] and are relatively slower and faster respectively than the preferred walking velocity of individuals with CFS (1.05 ms^-1^) already reported [8]. It is important to examine the joint kinematics at matched velocities to assess where any differences occur, and from a rehabilitation perspective, allow clinicians to plan more effective and focussed treatment programmes.

## Methods

### Subjects

Twelve individuals with CFS (aged 52.2 ± 11.3 years) and 12 age and sex matched control subjects (aged 52.8 ± 11.8 years) participated. The individuals with CFS were recruited from three local CFS support groups and had a diagnosis of CFS confirmed by their medical practitioner. The control subjects were a convenience sample of University staff and friends. For both the individuals with CFS and control group subjects were excluded from the study if they suffered from significant orthopaedic, neurological or cardiovascular problems which may affect their gait pattern. The mean height of the individuals with CFS and control group were 163.0 cm (± 9.2) and 166.0 cm (± 7.1) respectively and this difference was not statistically significant (p = 0.209). Similarly there were no statistically significant differences between the two groups in terms of body mass (controls 70.1 ± 7.4 kg and CFS 68.5 ± 10.9 kg; p = 0.644). The individuals with CFS had been suffering from the condition for an average of 13.6 years ± 4.5 years). From the SF-36, the mean physical functioning score of the CFS group was 27.9 (± 19.7) [9]. Of the 12 individuals with CFS 3 had taken early retirement due to their condition, 8 were unable to work and received state benefits and only one person was able to work part time. In terms of walking aids only three of the twelve individuals with CFS occasionally walked with a walking stick but no one in the control group required any walking aids. During the data collection none of the individuals with CFS used their walking aid.

### Ethical approval

The study was approved by South Glasgow University Hospital's Ethics Committee and all subjects gave written consent before participating in the study. All subjects were required to attend the Glasgow Caledonian University's Clinical Research Centre within the South Glasgow University Hospital for testing.

### Procedure

Each subject completed three successful trials at the three velocities; their preferred walking velocity, and then two controlled velocities: a slower velocity and a faster velocity. For the controlled velocities the subjects were expected to cover 7.2 m from a standing start and stopping after 16 and 5.4 seconds respectively, indicative of averaging walking velocity of 0.45 ms^-1 ^and 1.34 ms^-1^. The order of tests was the same for each subject (preferred, slow and then fast). A seat was positioned at the beginning of the walkway and all subjects, especially the individuals with CFS were encouraged to rest as required; between each test and/or each velocity. For the set velocities an audible tone was generated on a PC using PowerPoint (Microsoft Corporation) slide transition advance facility to signal the subject to begin walking and a further tone when the subject should have reached the end of the walk. Prior to data collection the subjects were given clear instruction, demonstration and practice if necessary of the gait velocity required. If the subject did not reach the end of the walkway as the finish tone occurred the trial was repeated.

Gait analysis was conducted using a seven camera Qualisys Motion Analysis System (Qualisys Medical AB, Esperantoplatsen 7–9, S-411 19 Gothenburg, SWEDEN). Subjects wore cycling shorts and spherical reflective markers were attached to the pelvis and lower limbs. The anatomical landmarks for marker attachment were the anterior superior iliac spine (ASIS), the greater trochanter (GT), the lateral femoral condyle (LFC), the lateral malleolus (LM) and the base of the fifth metatarsal (FM). Data were collected at 60 Hz and the system calibrated to collected a volume of 5.0 (sagittal plane – X axis – direction of travel) by 1.5 (Z axis – vertical) by 1.0 (Y axis – coronal plane) metres using Qualysis TrackManager. Only calibrations with average residuals of less than 1.5 mm in all cameras were accepted prior to data collection. Kinematic parameters were calculated using Visual 3D (Version 3.28) (C-Motion, Inc., 15821-A Crabbs Branch Way, Rockville, MD 20855, USA). Virtual markers were created for the medial femoral condyle (VMFC), medial malleolus (VMM), and first metatarsal (VFM) from anthropometric data taken from each participant. All marker data were low-pass filtered using a 4^th ^order Butterworth filter with cut-off frequency of 6 Hz, and interpolated with a maximum gap fill of 5 frames using a 3^rd ^order polynomial. The body segments were defined using the ASIS and GT for the pelvis; GT, LFC, and VMFC, with radius determined by anthropometic data from each subject, for the thigh; LFC, VFMC, LM, VMM for the shank; and LM, VMM, FM and VFM for the foot. Hip angle was defined as the Cardan (default setting for Visual 3D) representation between the proximal pelvis and distal thigh; knee angle between proximal thigh and distal shank; and ankle angle between proximal shank and distal foot. All proximal segments were considered as the reference segment. Joint angles were normalised to the joint angles during quiet standing (the angle measured at each joint during quiet standing were considered to be the joint in neutral and all subsequent measures expressed relative to that), collected for a duration of 1s before the gait collection for each subject. Only successfully interpolated data were included in the analysis. A successful trial was considered to be one which required no interpolation of the marker trajectories and no markers were obscured during collection. Most subjects completed this in their 1^st ^three trials at each velocity however 2 controls and 3 individuals with CFS required 4 trials (1 fast and 1 self selected, and 1 fast and two self selected respectively). Time events were generated from visual inspection of the modelled gait for initial contact (when the lateral malleolus became static in the X direction) and final contact (when the 5^th ^metatarsal started to move forward in the X direction) for both sides. Stance phase was defined as initial contact on one side to final contact on the ipsilateral side and swing phase was final contact to initial contact. Joint angles were calculated for stance and swing phases. All data were normalised in the time domain to 100% for each phase. One stride per side per trial for each velocity were averaged and sagittal peak to peak range of motion for stance and swing phases analysed.

### Data analysis

Data were checked and entered to Excel spreadsheets, then imported to the GenStat statistical package (GenStat Committee, Oxford 2005). The mean and standard deviation of each spatial and temporal parameter and each kinematic parameter were calculated for individuals with CFS and controls separately, at each of the walking velocities. A Manova (Multivariate analysis of variance) was carried out comparing individuals with subjects with CFS and controls. Manovas were performed on the following parameters. Step Distance; Step Time; Single and Double Support Time; and range of movement at hip; knee and ankle. Within each Manova results for both right and left sides were grouped, in addition for Manovas on kinematic data results for both stance and swing phases were grouped. This approach resolves many issues regarding multiple comparisons. The Manova performs two tests; Status of case-control (CFS versus control), of velocity (self-selected versus slower versus faster). If the Manova was non-significant then no further tests were performed. Where a Manova test yielded a significant result (P < 0.05) then paired t-tests were conducted on those variables. A difference between the two groups was regarded as statistically significant for the paired t-test if P < 0.05.

## Results

### Self-selected velocity

The mean self-selected velocity of the CFS and control groups was 0.99 ms^-1 ^(± 0.27 ms^-1^) and 1.32 ms^-1 ^(± 0.15 ms^-1^) respectively (P = 0.002), At the self-selected velocity there was a significant difference between the two groups for all the temporal and spatial variables (Table 1). These results were very similar to those previously reported [8].

**Table 1:** Temporal and spatial parameters of gait of both the individuals with CFS and control group. Temporal and spatial parameters of gait of both the individuals with CFS and control subjects at self selected pace, at the slower matched velocity and at faster matched velocity. R = Right side of the body and L = left side of the body. NS represents a non significant result from the MANOVA, reported P values are those calculated from resulting paired t-tests between individuals with CFS and controls.

	Self Selected			Slower matched velocity			Faster matched velocity		
Parameter	CFS	Control	p value	CFS	Control	p value	CFS	Control	p value

Velocity (ms^-1^)	0.99	1.32	0.002	0.46	0.48	NS	1.3	1.31	NS
Step distance R (cm)	55.8	64.9	0.037	42.1	46.1	0.003	65.3	64.9	NS
Step distance L (cm)	55.5	65.7	0.010	41.5	48.2	0.047	62.8	65.7	NS
Step time R (s)	0.57	0.50	0.002	0.92	0.97	NS	0.49	0.50	NS
Step time L (s)	0.58	0.51	0.002	0.92	0.99	NS	0.48	0.51	NS
Single support R (s)	0.44	0.39	0.003	0.62	0.66	NS	0.39	0.39	NS
Single support L (s)	0.45	0.39	0.010	0.63	0.63	NS	0.41	0.39	NS
Double support (s)	0.26	0.21	0.017	0.59	0.68	NS	0.19	0.21	NS
Cadence (steps/min)	105	120	0.001	68	63	NS	124	120	NS

Results of the MANOVAs and appropriate follow up paired t-test are presented in Table 2. Analysis of the kinematic variables at self-selected velocities indicated that the group mean joint excursion was generally less for individuals with CFS than the controls. The range of movement at the ankle during swing phase, for both sides, showed a significant reduction for the individuals with CFS in comparison to the controls. For the right ankle there was a significant, though marginal, reduction in range of movement during the stance phase (P = 0.049).

**Table 2:** Kinematic variables (degrees) of gait for both the individuals with CFS and control subjects. Kinematic variables (in degrees) of gait for both the individuals with CFS and control subjects at Self selected velocity, at the slower matched velocity and at the faster matched velocity. Results are given for both the right and left sides. All values given represent the group mean range of movement of each of the lower limb joints during both stance and swing phase. NS represents a non significant result from the MANOVA, reported P values are those calculated from resulting paired t-tests between individuals with CFS and controls.

	Self – selected velocity			Slower matched velocity			Faster matched velocity		
ROM (degrees) Right	CFS	Control	p value	CFS	Control	p value	CFS	Control	p value

Hip stance	35.2	39.4	NS	28.6	32.3	NS	39.1	39.4	NS
Hip swing	32.9	37.4	NS	25.5	30.7	NS	37.3	37.4	NS
Knee stance	22.1	22.1	NS	22.0	22.2	NS	23.5	22.1	NS
Knee swing	53.3	59.3	NS	47.4	53.7	NS	54.6	59.3	NS
Ankle stance	12.6	16.8	0.049	14.5	21.4	0.014	13.6	16.9	NS
Ankle swing	11.5	20.9	<0.001	11.9	19.4	0.001	14.6	20.9	0.001

ROM (Degrees) Left	CFS	Control	p value	CFS	Control	p value	CFS	Control	p value

Hip stance	38.0	40.2	NS	31.2	30.7	NS	41.3	40.2	NS
Hip swing	35.2	37.8	NS	27.1	30.3	NS	38.5	37.8	NS
Knee stance	22.3	22.5	NS	23.7	20.1	NS	22.1	22.6	NS
Knee swing	53.7	60.1	NS	49.1	53.8	NS	54.0	60.1	NS
Ankle stance	16.3	17.8	NS	18.2	20.5	NS	16.8	17.8	NS
Ankle swing	13.9	22.0	0.008	12.9	18.5	0.032	15.9	22.0	0.010

Thus it does appear that there are a number of significant gait differences between individuals with CFS and control subjects at their self-selected velocity. All temporal and spatial parameters showed a significant reduction, and although not significant for many of the kinematic parameters all but one showed a reduction in the range of motion for the individuals with CFS.

**Figure 1:**
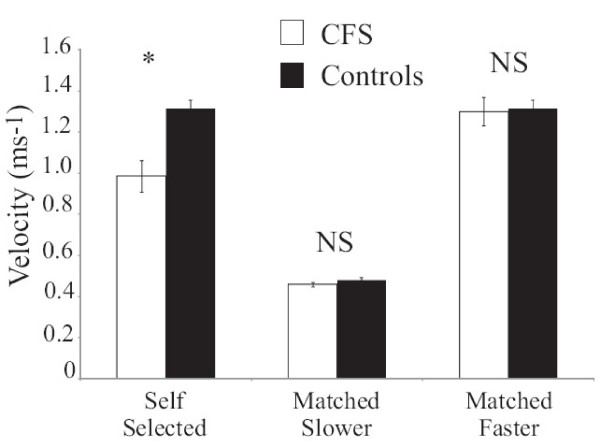
**The group means (and standard deviation) of the gait velocity obtained at the different velocities**. The actual group mean (and standard deviation) of the gait velocity obtained at each of the different testing velocities (self selected, slower matched velocity and faster matched velocity. CFS subjects are shown in black and controls in white. NS represents non-significant differences and * denotes a significant difference.

However as previously stated the self selected velocity of the individuals with CFS was significantly slower than that of their matched control and, as many gait parameters are dependent on velocity it was important to compare subsequently the two groups at matched velocities [10-13]. The protocol used in this study aimed to compare the gait patterns at a slower walking velocity (0.45 ms^-1^) and a faster velocity (1.34 ms^-1^). However although the average walking velocity along the total walkway was close to the desired velocity it can be seen from Figure 1 that, when the data were captured i.e. around the middle of the 7.2 m walkway, there was an obvious difference in walking velocity between the two groups at the faster velocity. Any differences which were found between the individuals with CFS and controls group at this faster velocity may have been a reflection of the difference in velocity. Therefore it was decided only to analyse the slower velocity, and also to compare the individuals with CFS at the faster walking velocity with the controls at their self-selected walking velocity, where both groups were closely matched in terms of walking velocity.

### Matched Velocities

There was no statistical difference in walking velocity between the two groups at the slower velocity (P = 0.120). In terms of the temporal and spatial parameters, the only statistical difference between the two groups, CFS and controls, was a reduction in the step distance of both right and left sides. There were no statistical differences observed in any of the other temporal and spatial gait parameters (Table 1). With regards the kinematic results the pattern of differences between the two groups was similar to that observed at the self selected velocity i.e. a reduction in the group mean range of movement of the right ankle during both swing and stance phases and the left ankle during swing phase (Table 2).

There was no statistical difference in walking velocity when comparing the individuals with CFS at the faster velocity (1.30 ± 0.24 ms^-1^) and the Controls at their self-selected walking velocity (1.32 ± 0.15 ms^-1^) (p = 0.781). No statistical differences were observed for any of the temporal and spatial parameters (Table 1). For the kinematic data the only statistical differences were observed as a reduction in the range of movement of both ankles during the swing phase (Table 2).

Thus overall the results of this study suggest that, at self-selected velocity, the gait pattern of those with CFS is quite different to that of healthy controls but many of the differences observed may be a direct result of the relatively slow self-selected gait velocity of the individuals with CFS. When the walking velocities of the two groups were matched during a relatively slow gait velocity there were fewer differences in the temporal, spatial parameters. More importantly, however, when the individuals with CFS subjects were matched to a more 'normal' gait velocity, the two groups displayed a similar gait pattern which suggests that the observed differences between the groups at self-selected velocity may have been primarily a reflection of the relatively slow walking velocity of the individuals with CFS. The range of ankle motion during the swing phase of gait was the only kinematic consistently lower for individuals with CFS regardless of the velocity of the walk.

## Discussion

One of the most obvious results of the present study was a statistically significant difference in the self-selected walking velocities of the CFS and control groups. Indeed the CFS group exhibited an average self-selected walking velocity of 0.99 ms^-1 ^(SD ± 0.27) which is below the normal walking velocity of around 1.2–1.4 ms^-1 ^and is comparable to the walking velocity of above knee amputees [14]. The differences appear to be mainly in the temporal and spatial parameters with the CFS subjects taking smaller and slower steps compared to the controls. These temporal and spatial differences are consistent with those previously reported [8]. Kinematic data suggest the altered gait pattern may be a result of reduced range of movement of the lower limb joints, although not significant other than ankle range of motion during swing phase for both sides and marginally during stance for the right side, cumulatively these reductions in range of motion at the joints result in an altered gait pattern. This study confirms previous work that those who suffer from CFS have an altered self selected gait pattern.

The cause of the gait differences cannot be inferred from the present study however work to investigate this is currently underway. Chronic Fatigue Syndrome has a complex presentation, characterised by a variety of physical signs and symptoms which may alone, or in combination, affect the gait pattern of those with CFS. For example pain may be a significant factor affecting the way people with CFS walk. Very little is known about the pain pattern of those with CFS and, critically for the present study, whether it follows a symmetrical or asymmetrical presentation. Boda et al. [4] proposed that the gait differences they observed between CFS subjects and controls could be due to altered balance mechanisms, peripheral neuromuscular dysfunction and/or neurological abnormalities in those with CFS. It would seem reasonable that any of these factors could explain the differences we observed. For example Sieminonow et al. [15] reported a greater level of cortical activation required to undertake voluntary tasks for those with CFS compared to healthy subjects. The increased effort required for walking in those with CFS might lead to greater central contribution to muscle fatigue and may explain the differences in step length between the CFS and control subjects. One way to investigate this central contribution to fatigue may be to monitor changes in spinal motoneuronal activity following fatiguing exercise in the CFS group, and this is currently being undertaken by our group.

As already stated, the self-selected gait velocity was significantly lower in the CFS group compared to the control subjects. It is likely that the individuals with CFS adopt a slower self selected walking velocity to reduce their energy expenditure when walking however although the energy expenditure is reduced it is known that slower walking speeds are less efficient and that overall the metabolic cost of walking increases at slower, and also faster, walking velocities [14,16,17]. Thus the slower self-selected velocity may in itself increase the overall effort required for normal walking in those with CFS. Investigating the physiological cost of walking is relatively straightforward with current gas analysis equipment and our group are currently investigating the physiological cost of over-ground walking in CFS sufferers as a follow up to the present study.

When the walking velocity was matched between the two groups at the slower velocity (0.45 ms^-1^) it was found that the only difference in the temporal and spatial parameters was the step distances on both sides. Furthermore the kinematic profile at matched (slow) walking velocities was very similar to the data obtained at the self-selected velocity in that the differences were observed in the range of movement of the ankle during both the stance (right side only) and swing phases of gait. There are very few studies which have examined the gait patterns of subjects with CFS. Boda et al [4] examined CFS and control subjects walking on a treadmill at the same slow walking velocity used in the present study (0.45 ms^-1^). They reported that the CFS group utilised shorter steps than the controls and this is consistent with the results of the present study. They suggested that this difference was due to reduced flexion at both hips and knees of the CFS group. However, the kinematic results of the present study found differences only at both ankles during swing and at the right ankle during stance. These differences in the kinematic parameters between the two studies may be related to the fact that subjects in the study by Boda et al. [4] were walking on a treadmill whereas in the present study the subjects were walking over-ground. Whilst the debate over the association between the gait pattern of over-ground and treadmill walking continues [5,18] it is true that one of the main advantages of the treadmill is that walking velocity can be more accurately standardised and therefore matched between subjects. In the current study we were unable to directly compare subjects at the faster velocity (1.34 ms^-1^) as the achieved gait velocity was statistically different between the two groups. Treadmill walking would have allowed better control of faster walking velocities but may have changed the natural gait pattern which we wished to observe.

These results therefore appear to suggest that there are gait differences between the CFS and control group and this may be due to the factors already discussed in relation to self-selected velocity. However this comparison was made at a relatively slow walking velocity (0.45 ms^-1^) which would not reflect normal activity.

Perhaps the key finding of the present study was that when performing a more functionally relevant comparison: that of the control subjects at their self selected velocity to the CFS subjects at their faster walking velocity (which represented a 'normal' velocity) results revealed very similar gait patterns between the two groups. The only parameter which showed a statistically significant difference was the ankle range of movement during swing for both legs which may suggest peripheral muscle weakness although this cannot be specifically inferred from the present results. Thus it appears that this sample of CFS subjects are able to walk at a 'normal' gait velocity, with a 'normal' kinematic gait pattern but for whatever reasons they do not do so.

As with many studies in this area one of the main limitations is the small sample recruited for the study. Individuals in the CFS group were not specifically asked if they also had fibromyalgia, a condition with many overlapping symptoms to CFS. Pain is the primary feature of fibromyalgia and may have affected the gait in some individuals, however the presence or extent of gait abnormality in those with fibromyalgia is unknown.

## Conclusion

It appears that those with CFS exhibit an altered gait pattern compared to healthy controls at self-selected velocity confirming previous studies and clinical reports of altered gait in CFS. However when CFS subjects increase their walking velocity they are able to attain a more 'normal' gait pattern for sagittal kinematic and temporal-spatial parameters. Further research is required to investigate the underlying cause of these gait differences in CFS and the physiological cost and kinetics of walking at self-selected and matched velocities in order that therapeutic interventions can be effectively implemented to encourage a more normal and efficient gait pattern in this group of people.

## Competing interests

The authors declare that they have no competing interests.

## Authors' contributions

LP contributed to the design, data collection, clinical relevance, and analysis of the data presented.

DR contributed to the design, data collection, technical aspects of the measurements, and analysis of the data presented.

LW contributed to the design, data collection, physiological interpretation, and analysis of the data presented.

WMacL contributed to the design, and statistical analysis of the data presented.

All Authors have read and approved final manuscript
